# Impact of multimorbidity on risk and outcome of stroke: Lessons from chronic kidney disease

**DOI:** 10.1177/1747493020975250

**Published:** 2020-11-27

**Authors:** Dearbhla M Kelly, Peter M Rothwell

**Affiliations:** Nuffield Department of Clinical Neurosciences, Wolfson Centre for Prevention of Stroke and Dementia, John Radcliffe Hospital, University of Oxford, UK

**Keywords:** Multi-morbidity, stroke risk, chronic kidney disease, stroke, transient ischemic attack, hypertension

## Abstract

With both an aging population and greater post-stroke survival, multimorbidity is a growing healthcare challenge, affecting over 40% of stroke patients, and rising rapidly and predictably with increasing age. Commonly defined as the co-occurrence of two or more chronic conditions, multimorbidity burden is a strong adverse prognostic factor, associated with greater short- and long-term stroke mortality, worse rehabilitation outcomes, and reduced use of secondary prevention. Chronic kidney disease can be considered as the archetypal comorbidity, being age-dependent and also affecting about 40% of stroke patients. Chronic kidney disease and stroke share very similar traditional cardiovascular risk factor profiles such as hypertension and diabetes, though novel chronic kidney disease-specific risk factors such as inflammation and oxidative stress have also been proposed. Using chronic kidney disease as an exemplar condition, we explore the mechanisms of risk in multimorbidity, implications for management, impact on stroke severity, and downstream consequences such as post-stroke cognitive impairment and dementia.

## Introduction

The term “multimorbidity” is typically defined as the presence of two or more long-term conditions within an individual.^1^ Due to an increasing life expectancy, there is now a substantial and growing global burden of multimorbidity that affects low- and middle-income countries (LMICs) as well as high-income ones.^2^ In LMICs, rates of non-communicable diseases such as diabetes and cardiovascular disease are rising and augmenting existing burdens of infectious diseases, maternal and child health problems, and nutritional conditions.^3,4^ However, research on multimorbidity has many evidence gaps and there have been several calls to make it a priority for global health research.^2,5,6^ In this review, using chronic kidney disease (CKD) as an exemplar condition, we will explore the current knowledge of and evidence for the impact of multimorbidity on stroke risk, care, and outcomes. CKD is a good example of this phenomenon because, as we will demonstrate, it is under-recognized, increasing in prevalence, associated with both traditional vascular risk factors as well as non-traditional ones, and it can potentially impact every stage of stroke presentation, diagnosis, treatment, and outcome.

### Search strategy

The methodology of our systematic reviews of stroke and CKD has previously been published.^7,8^ Briefly, we searched MEDLINE and EMBASE databases (from inception to May 2020) using a search strategy developed by a specialized librarian that combined text word and medical subject headings without language restrictions. Additionally, we searched references from relevant articles. The final reference list was generated based on relevance for the current review.

## Epidemiology and implications of multimorbidity in stroke

There has been variation in the duration, severity, or clustering of conditions included in the definition of multimorbidity which can make direct comparison of studies or populations challenging.^9^ In a nationwide population-based cohort study of 219,354 Danish adults hospitalized for first stroke, there was a 42.7% prevalence rate for multimorbidity as measured using the Charlson Comorbidity Index (CCI).^10^ The most prevalent comorbidities were atrial fibrillation (AF) or flutter (11.0%), cancer (10.9%), diabetes mellitus (9.0%), congestive heart failure (8.1%), and chronic pulmonary disease (8.1%). The association between multimorbidity and stroke predictably appears to increase with age. In a smaller, retrospective cohort study (*N* = 29,673) of community-dwelling older (≥66 years) adults living with stroke in Ontario, Canada, 99.1% had one or more comorbidities, 51.7% had four or more, and 6.5% had seven or more comorbidies.^11^ In this study, although concordant conditions such as hypertension (89.9%) and ischemic heart disease (38.1%) were quite common, so too were discordant conditions such as arthritis (65.8%) and inflammatory bowel disease (21.4%). However, multimorbidity in younger stroke survivors is also an issue in certain high-risk populations or geographic regions such as in the rural areas of Appalachian states in the US where there was a 21% prevalence of multimorbidity in those aged less than 50 years.^12^

Despite its high prevalence, the impacts of multimorbidity on stroke severity, functional recovery, and long-term risk of recurrence are not clearly understood. Certain specific comorbidities such as AF and diabetes are well known to be associated with poor functional outcomes and worse responses to acute therapies such as thrombolysis.^13,14^ However, few large-scale studies have examined the relationship between multimorbidity and functional outcomes post-stroke and those that have report variable findings.^15,16^ A French study of 28,201 stroke survivors found that the CCI was associated with less functional gain, even after adjusting for baseline functional status and length of stay.^15^ In contrast, a smaller US study (*N* = 2402) reported that CCI did not improve the predictive model fit for functional independence over age/sex alone. However, this study did find that diagnostic cost group, another measure of multimorbidity, improved the overall model fit for functional gain, suggesting that method of measurement may matter. It has been recently highlighted that there are at least 35 objective measures of multimorbidity available and that each of these uses different variables to generate a score or index, linked with various or no outcome measures.^17^ The best measure of multimorbidity in the context of stroke is unknown.

The impact of multimorbidity on risk of stroke recurrence as well as subsequent vascular risk is also poorly described. However, several analyses from the Oxford Vascular Study (OXVASC), an ongoing population-based study of all types of acute vascular events (including transient ischemic attack (TIA), stroke, acute coronary syndromes, and peripheral vascular events) in the UK since 2002, have tried to provide some insights in this area.^7,18–20^ First, it has been shown that among 2554 TIA/stroke patients with over 10,000 patient-years of follow-up, the number of affected vascular beds (cerebrovascular, coronary, peripheral vascular) predicted the long-term risk of both recurrent stroke and non-stroke vascular events.^18^ Compared with patients with single-territory, patients with multiple-territory disease also had higher long-term risks of recurrent ischemic stroke (hazard ratio (HR) = 1.38; 95% CI: 1.04–1.81) and non-stroke acute vascular events (HR = 3.06; 95% CI, 2.23–4.20). Second, the Essen score, a simple clinical score based on the presence of prior vascular comorbidities, was used to risk-stratify OXVASC patients with TIA/stroke without prior coronary artery disease (CAD) and to identify subsets who may be at high risk of further vascular events.^19^ Compared with patients with prior CAD, an Essen risk score of ≥4 identified a subgroup at similar high 10-year risks of myocardial infarction and of recurrent stroke. Third, OXVASC has highlighted that patients with TIA/stroke with coexisting cardiovascular disease remain at high risk of recurrent ischemic events despite standard secondary prevention treatment.^20^ However, the predictive value of non-vascular comorbidities or overall multimorbidity scores for recurrent vascular events is less clear.

Multimorbidity is also consistently associated with short-term and long-term mortality post-stroke.^10,21–23^ In the large Danish national registry-based study, there was a dose-response relationship between multimorbidity (as measured by CCI) and 30-day and 5-year mortality risk over the 18-year follow-up period.^10^ Stroke survivors with very severe comorbidities (defined as a CCI score of ≥3) had a 23.5 and 74.5% 30-day and 5-year mortality risk, respectively, compared with 10.5 and 36.6% for those without comorbidities at baseline. Comorbidities such as cancer and advanced renal or liver disease are associated with a particularly high mortality post-stroke^24^ beyond the combined expected effects of comorbidity and stroke acting alone though other studies suggest that it is the number of comorbidities rather than the type of condition that may be the more helpful predictor of mortality.^23^ This association is thought to be multi-factorial, variously attributable to greater stroke severity and subsequent disability,^25^ polypharmacy,^26^ bleeding diastasis, or hypercoaguloability.^27^

In a Canadian cohort study of older individuals with stroke, increasing number of comorbidities was associated with greater healthcare utilization (primary and secondary care, emergency department, home care visits, and hospitalizations) during the five years’ post-stroke.^11^ The authors of this study highlighted that, in 2008, for example, the average older adult with prior stroke visited a primary care physician over 13 times not including specialist visits, albeit for predominantly non-stroke reasons. Health care costs increased accordingly with multimorbidity burden, mainly driven by the increase in acute care use.

However, there are some limitations to the concept of multimorbidity in stroke that should be considered. First, the approach that is sometimes used of simply measuring the number of comorbidities is arguably a simplistic concept that fails to appreciate the spectrum/severity of each disease and assumes that each comorbidity has an equivalent impact if using a simple quantitative approach. Second, multimorbidity may potentially overestimate disease burden when overlapping conditions (or conditions on the same causative pathway) exist. Finally, the multimorbid approach may not consider the cumulative effect on physiology and functional reserve (Fried’s clinical frailty phenotype).^28^ Although multimorbidity and clinical frailty are separate concepts, they frequently co-exist and a consideration is that it is not necessarily multimorbidity but the associated frailty that may be driving worse outcomes.

## CKD – An exemplar comorbidity

CKD exemplifies the complexity of multimorbidity and its multifaceted impact on stroke, from etiology to rehabilitation. Although CKD itself is a heterogenous condition that encompasses a variety of diseases including diabetic or hypertensive nephropathy, glomerulonephritidies, polycystic kidney disease, amongst others, it is consistently associated with cardiovascular disease risk and burden.^29^ In this review, we will explore the pathways through which it leads to stroke, and how it impacts on treatment and outcomes.

**Figure 1.**
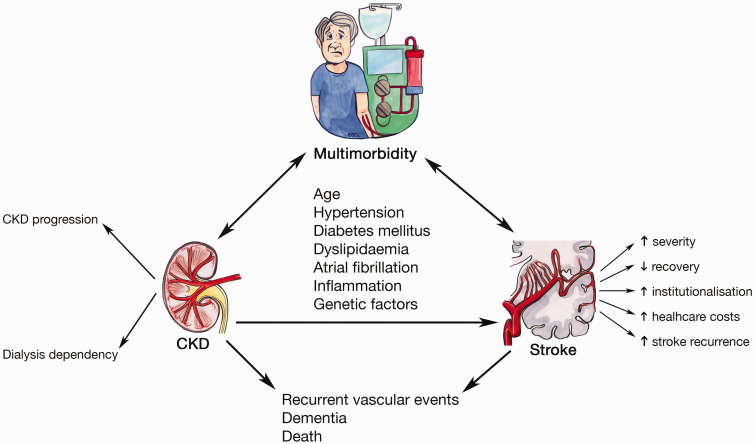
The interplay of CKD, stroke, and multimorbidity.

CKD is often under-recognized as an important and frequent comorbidity that affects stroke survivors (Figure 1). This is partly because patients with kidney disease have been excluded from over one-third of clinical trials of cerebrovascular disease interventions and only 0.3% of trials have subsequently reported baseline renal function.^30^ In addition, many of the existing multimorbidity scores or measurement tools do not take CKD into account or acknowledge only severe disease as in the case of the CCI,^31^ the most commonly used tool in studies of stroke and multimorbidity.^1^ However, this is an important omission since even early stages of CKD are associated with cardiovascular mortality^32^ and CKD itself frequently portends other multimorbidity.^33^

The global burden of CKD is on the rise with prevalence estimates of 9.1% (8.5–9.8) for all stages.^34^ However, CKD prevalence is much higher in acute stroke survivors, varying from 20 to 40% in patients with acute ischemic stroke^35,36^ and from 20 to 46% in patients with acute intracerebral hemorrhage (ICH).^37,38^ Kidney function, as determined by the estimated glomerular filtration rate (eGFR), demonstrates an inverse step-wise relationship with incident stroke risk increasing by 3-, 4.1-, 5.4-, and 7.1-fold for CKD stages 3–5 and dialysis compared to the general population.^39^

Patients with proteinuria appear to represent an even higher-risk subgroup within kidney disease. A previous meta-analysis showed that proteinuria was associated with a 70% increased risk of stroke compared to those without it,^40^ and other studies have suggested that it may be a better predictor of stroke risk in CKD than low eGFR.^41^ There is also some evidence though that stroke risk increases additively with declining GFR and increasing albuminuria.^42^ Proteinuria itself has been associated with an independently increased risk of multimorbidity.

## Mechanisms of risk in CKD

Disentangling the pathophysiology of stroke risk in multimorbidity can be challenging as although shared vascular risk factors may account for much it, novel risk factors have also been proposed and their role has yet to be fully elucidated (Figure 1). For example, it has been postulated that stroke risk in CKD may be attributable to a combination of both traditional and non-traditional cardiovascular mechanisms.^43^ These can be further subdivided into shared conventional vascular risk factors such as hypertension and diabetes, secondary consequences of renal dysfunction such as chronic inflammation and mineral-bone disease, dialysis-specific factors such as cerebral hypoperfusion, and systemic conditions that cause both stroke and CKD such as systemic lupus erythematosus or Fabry’s disease.^44^

The relevance of non-traditional risk factors gained some traction when earlier meta-analyses found that CKD (defined an eGFR below 60 mL/min/1.73 m^2^) appeared to independently increase the risk of incident stroke by 43% and that stroke risk increased 7% for every 10 mL/min/1.73 m^2^ decrease in eGFR.^42,45^ These associations were consistent across subtypes of stroke, sex, and varying prevalence of cardiovascular risk factors.

**Figure 2.**
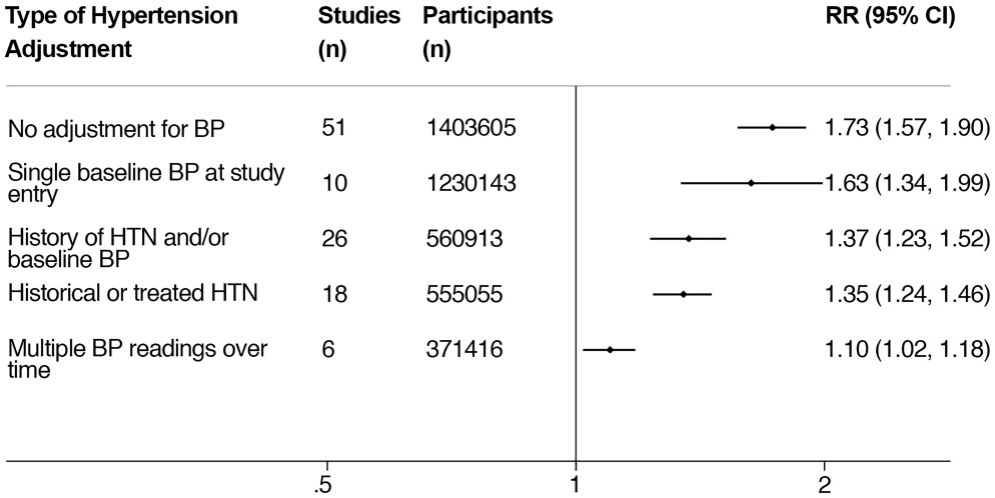
Variation in the risk association between CKD and stroke depending on the method of hypertension adjustment used in the studies. All studies were also adjusted for other traditional risk factors. Reproduced from Kelly et al.^7^ CI: confidence interval; BP: blood pressure; HTN: hypertension; RR: relative risk.

However, apparent associations between comorbidities and stroke risk can be complex. For example, the relationship between CKD and cerebrovascular disease might not be truly independent of hypertension, the most prevalent comorbidity in individuals with CKD,^46^ and the leading modifiable risk factor for stroke in the general population.^47^ In a systematic review and meta-analysis of stroke risk with low eGFR (<60 mL/min/1.73 m^2^) with a particular focus on how robustly studies adjusted for hypertension,^7^ 85 studies were included in which 3,417,098 participants experienced nearly 73,000 stroke events. Although patients with CKD appeared to have a 36% greater risk of stroke than in those with normal renal function in multivariate-adjusted analysis, this risk association varied considerably depending on the way in which hypertension was adjusted for (Figure 2). When multiple prior BP readings over time were adjusted for, a better marker of long-term burden or control, there was near-complete attenuation of the risk association between CKD and stroke. The degree of risk attenuation would tend to suggest that this relationship is strongly confounded by hypertension and that CKD, as determined by low eGFR, is unlikely to be a significant independent risk factor for stroke outside of such traditional risk factors.

**Figure 3.**
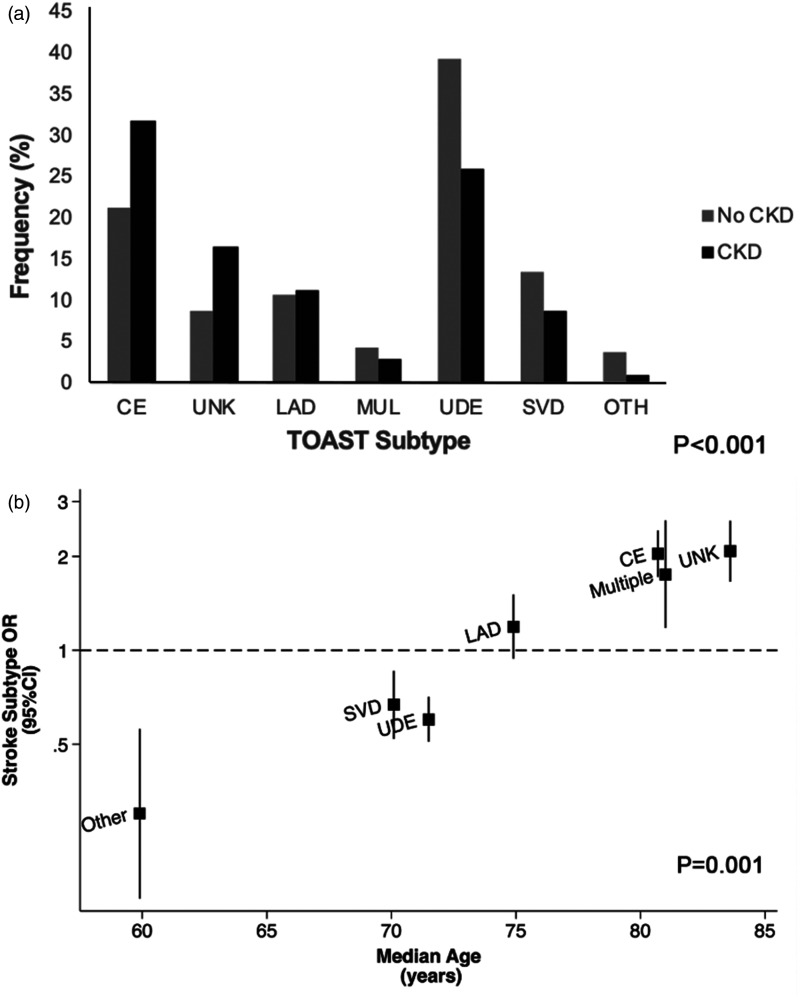
(a) CKD initially appeared to be associated with certain TOAST TIA/stroke subtypes; (b) however, these associations were shown to be strongly confounded by age when the odds ratio (OR) of specific TOAST subtypes in CKD was plotted against the median age within individual subtypes. Age was a significant predictor of between-subtype variance in a metaregression. Reproduced from Kelly et al.^36^

Confounding by age is also an issue that must be carefully teased out when examining the relationship between stroke and comorbidities. CKD is particularly liable to this problem since its GFR-based definition (60 mL/min/1.73 m^2^ for three or more months as per 2012 Kidney Disease: Improving Global Outcomes guidelines)^48^ is very age-dependent in its measurement. For example, approximately one-half of adults over the age of 70 years have a measured or eGFR <60 mL/min/1.73 m^2^.^49^ There has been controversy over whether mildly reduced eGFR in this context truly represents disease versus the normal structural and functional changes that occur in the kidney with ageing.^50^ Thus, although there appears to be major differences in rates of CKD between etiological (TOAST) stroke subtypes, for example, in a study of nearly 3000 patients with TIA and ischemic stroke, the association was strongly confounded by age (Figure 3).^36^ Any study of multimorbidity and stroke risk should therefore be carefully stratified by and adjusted for age.

Chronic inflammation or “inflammageing,” a condition thought to occur in some older individuals, characterized by elevated levels of blood inflammatory markers that increases susceptibility to cardiovascular disease, has also been proposed to explain some of the association between CKD and stroke.^51^ However, in an analysis of nearly 1300 TIA/stroke patients, correlations between biomarkers related to inflammation and thrombosis with renal dysfunction in the setting of cerebrovascular events were generally modest after adjustment for age, suggesting that putative risk factors such as chronic inflammation or coagulopathy are unlikely to be important stroke mechanisms in patients with CKD.^52^

However, mechanisms of stroke risk may be more complex and less confounded for patients with proteinuria. The “strain vessel hypothesis” has been proposed whereby juxtamedullary afferent arterioles in the kidney and cerebral perforating arteries in the brain are both small, short vessels exposed to the large pressure gradients and therefore most vulnerable to hypertensive vascular injury, manifest as albuminuria and cerebrovascular disease, respectively.^53^ In a systematic review and meta-analysis of nearly 2 million participants undertaken to study the impact of hypertension on the relationship between proteinuria and stroke risk,^8^ the presence of proteinuria conferred a 70% greater risk of stroke compared to that in those without it. However, in contrast to low eGFR and stroke, the risk association between proteinuria and stroke did not substantially vary or attenuate even with the extensive adjustment for blood pressure. The association was also stronger in younger populations suggesting that shared genetic susceptibility for premature vascular disease may underpin the relationship between proteinuria and stroke risk.

## Inequities and implications for clinical practice

The high prevalence of CKD poses unique challenges to diagnosis, management, and prevention in stroke (Figure 1). In terms of presentation, it has been shown that in over one-third of cases, dialysis-dependent patients may develop stroke symptoms during or within 30 minutes of a dialysis session,^54^ which can lead to misdiagnosis and delayed presentation.^55^ In a cross-sectional analysis of 148 end-stage kidney disease (ESKD) patients without a prior diagnosis of stroke or TIA, 46 (36.5%) had experienced one or more stroke symptoms.^56^ These unreported symptoms were then subsequently associated with a two- to threefold higher odds of cognitive or functional impairment, suggesting that these may have represented clinically significant but undiagnosed events.

Compounding the problem of delayed presentation is that when patients with CKD eventually do present, there may then be further challenges to surmount in terms of diagnostic imaging. The administration of iodinated contrast material, as required for computed tomography angiography, has been associated with acute kidney injury (AKI), termed historically “contrast-induced nephropathy (CIN),”^57^ and more recently, “contrast-associated AKI (CA-AKI).”^58^ Although it is increasingly recognized that even patients with prior kidney disease are at very low risk of CA-AKI in this context,^59^ CKD is still associated with treatment delays in the receipt of thrombolysis therapy.^60^ In addition, gadolinium-based contrast agents have also been associated with nephrogenic systemic fibrosis, a debilitating and relentlessly progressive skin condition, in patients with advanced CKD, precluding their use in this group.^61^

CKD patients have consistently been shown to receive suboptimal acute stroke care at every level. They are less likely to be prescribed aspirin acutely,^62^ to be thrombolyzed,^62,63^ or to be admitted to an acute stroke unit.^62^ The reasons for these differences are unclear and likely myriad, related to delayed presentation, real or perceived bleeding risk, premorbid functional status, and poor integration of stroke with renal care. Regardless, they should be explored and carefully delineated. Therapies such as thrombolysis and mechanical thrombectomy are also variably associated with higher symptomatic ICH and mortality rates in this group,^64–66^ and the excess mortality is often attributable to non-vascular causes such as pneumonia or sepsis, which likely stem from or relate to their multimorbidity burden.^67,68^ Several studies have also shown that patients with CKD are less likely to receive standard secondary preventative therapies or advice including smoking cessation, antiplatelet, or statin treatment.^63,69^ These inequities appear to worsen with declining renal function^63^ and are exacerbated by co-existing racial/ethnic disparities. In a study of 56,587 ESKD hemodialysis patients with AF, black, Hispanic, or Asian patients were more likely to experience stroke (13, 15, and 16%, respectively) when compared with white patients, but were less likely to fill a warfarin prescription (10, 17, and 28%, respectively).^70^

## Impact of CKD on stroke severity and outcomes

Unsurprisingly then, CKD as a comorbidity has important implications for stroke outcomes. In a recent analysis of 3178 patients, CKD was shown to play a major role in stroke severity and recurrence risk.^71^ CKD was independently associated with greater risk of ischemic stroke compared to TIA (adjusted OR = 1.31, 95%CI = 1.11–1.56; p = 0.002) and with greater initial NIHSS (adjusted OR = 1.28, 1.04–1.46; p = 0.018), driven mostly by those with advanced CKD (defined as an eGFR < 30 mL/min/1.73 m^2^) (adjusted OR = 2.59, 1.44–4.66; p = 0.001 for ischemic stroke; adjusted OR = 4.06, 2.04–8.06; p < 0.001 for initial NIHSS). Among patients with ischemic stroke, CKD was also associated with higher one-month mRS scores (adjusted OR = 1.40, 1.13–1.74; p = 0.002), similarly driven by those with an eGFR < 30 mL/min/1.73 m^2^ (adjusted OR = 6.51, 3.04–13.97, p < 0.001).

Consistent with these findings, in an analysis of the Get With The Guidelines (GWTG)-Stroke cohort, an eGFR < 30 mL/min/1.73 m^2^ was associated with greater odds of institutionalization.^72^ Interestingly, the risk of institutionalization was greatest for those with an eGFR < 15 mL/min/1.73 m^2^ who were not on dialysis as this often identifies a multimorbid group, previously deemed unfit for dialysis with poor health status at baseline. The combination of CKD and stroke therefore has substantial socioeconomic costs. Among Medicare patients in the US, the combination of stroke and CKD was the most costly chronic condition dyad and comprised four of the top five most costly triads of conditions.^73^

**Figure 4.**
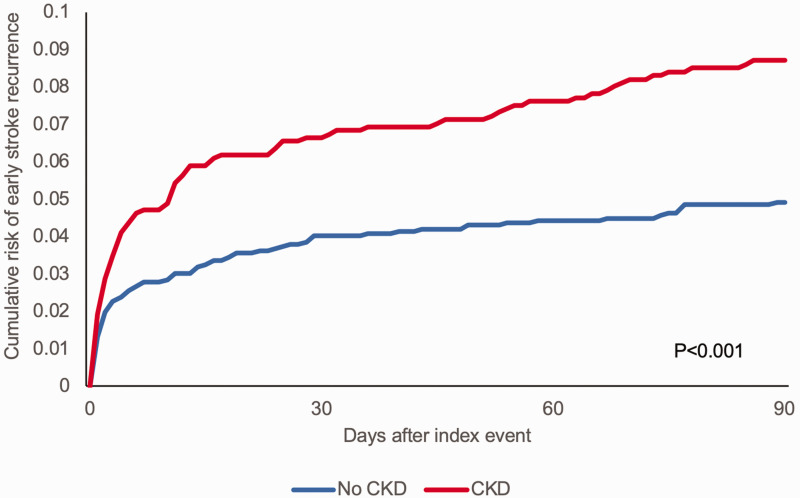
Cumulative risk of early (<90 days) recurrent stroke in those with CKD versus those with normal renal function. Reproduced from Kelly et al.^71^

In recent analysis,^71^ CKD was also independently associated with an increased risk of recurrent stroke (adjusted HR = 1.28, 1.05–1.55, p = 0.012), which was particularly pronounced for early (<90 days) stroke recurrence (adjusted HR = 1.60, 1.15–2.21; p = 0.005) (Figure 4). The sequential and consistent impact of CKD on initial event severity, early disability, and recurrence risk suggests that there may be associated inflammatory or other processes intrinsic to CKD leading to uniformly worse outcomes. One such unifying mechanism may be nitric oxide deficiency which is known to occur in CKD.^74^ Nitric oxide has a crucial role in angiogenesis after ischemic stroke,^75^ and the associated collateralization is predictive of post-stroke neurological outcomes.^76^ It is also a cerebral and systemic vasodilator, modulator of vascular and neuronal function, and inhibitor of apoptosis.^77^ Nitric oxide donors are candidate treatments for acute stroke. Although they showed some promise in preclinical studies,^78^ any efficacy in clinical trials has not been demonstrated.^79^ However, it is not known whether such novel drugs may be specifically beneficial in an already nitric-oxide-deficient group such as CKD patients.

Consistent with multimorbidity in general, CKD has also been associated with both short- and long-term mortality post-stroke.^80,81^

## Impact of prior stroke on CKD outcomes

There is a two-way relationship between stroke and comorbidities such as CKD in terms of risk, morbidity, and mortality. In an analysis of the Salford Kidney Study, a UK prospective cohort of more than 3000 patients, patients who suffered a stroke prior to recruitment had worse clinical outcomes (death, progression to ESKD, and non-fatal cardiovascular events) than those without stroke during follow-up even after accounting for other known risk factors.^69^ Patients with a history of stroke were also less likely to commence dialysis despite having a higher rate of ESKD, suggesting that their index stroke may have rendered them less likely to tolerate dialysis.

The impact of stroke on renal progression was also demonstrated in a large Taiwanese population-based cohort study (*N* = 100,353) where stroke was associated with higher risks for incident CKD (adjusted HR = 1.43, 1.36–1.50; p < 0.001), decline in renal function (aHR = 1.22; p = 0.04), and progression to ESKD (adjusted HR = 1.30; p = 0.008). Subgroup analysis showed that it was younger stroke patients (<50 years) (aHR 1.61, p < 0.001) and those with concomitant diabetes mellitus (aHR 2.12, p < 0.001), hyperlipidemia (aHR 1.53, p < 0.001), or gout (aHR 1.84, p < 0.001) who were at higher risk of developing incident CKD, again highlighting the interplay of concordant comorbidities within stroke, and the ability of multimorbidity to transcend age. With its known association with accelerated atherosclerosis,^82^ CKD may indeed be a harbringer of multimorbidity at younger ages.

## CKD, subclinical cerebrovascular disease, and post-stroke cognitive dysfunction

With aging populations, co-occurrence of neurodegenerative and vascular pathologies is increasingly recognized.^83,84^ Chronic cerebral inflammation due to vascular risk factors exposure and genetic modulators (apoE4) has been proposed to increase beta-amyloid (Aβ) production while chronic small vessel disease (SVD) and vascular inflammation may drive inefficient perivascular and cell-mediated Aβ clearance.^85^ In addition, increased periventricular white matter hyperintensities are associated with elevated cerebral amyloid independent of confounders such as age, APOE genotype, and vascular risk factors.^86^

CKD has been strongly associated with SVD including white matter lesions,^87^ silent cerebral infarctions (SCI),^88^ perivascular spaces,^89^ and cerebral microbleeds.^90^ Strikingly, over half of patients with CKD or ESKD have been shown to have SCI.^91,92^ This association may simply be related to their shared vascular risk factors such as hypertension and diabetes,^93^ but it has also been proposed that SVD and CKD may be part of a multi-system small vessel disorder.^94^ This theory was explored in an analysis of 1080 consecutive patients with TIA/stroke who underwent MRI imaging.^95^ CKD was found to be associated with total SVD burden (as measured by the total SVD score) (OR = 2.16, 1.69–2.75; p < 0.001), but only at age <60 years (<60 years: OR, 3.97, 1.69–9.32; p = 0.002; 60–79 years: OR = 1.01, 0.72–1.41; p = 0.963; ≥80 years: OR = 0.95, 0.59–1.54; p = 0.832). These findings indicate that there is an age-specific association between CKD and SVD that is predominantly seen at younger ages and that may be explained by a shared genetic susceptibility. Two genome-wide association studies have indicated genetic pleiotropy between kidney and cerebrovascular disease, particularly with large artery atherosclerotic and small vessel stroke.^96,97^ At older (>60 years) ages, however, much of the association appears to be confounded by age and other vascular risk factors.

However, regardless of etiology, SVD has many implications for patients with CKD. In a small study of nearly 200 hemodialysis patients, the presence of SCI independently predicted future cerebrovascular and all-type vascular events (HR for cerebral events 7.33, 95% CI 1.27–42.25: for vascular events 4.48, 95% CI 1.09–18.41).^91^ Similarly, in another small cohort study of 142 CKD patients, SCI also heralded the composite outcome of renal progression and death from cardiovascular causes (HR, 2.16; 95% CI, 1.01–4.64; p = 0.04).^98^

CKD is associated with cognitive impairment, particularly with declining renal function.^99^ In the Reasons for Geographic and Racial Differences in Stroke (REGARDS) Study, each 10 mL/min/1.73 m^2^ decrease in eGFR < 60 mL/min/1.73 m^2^ was associated with an 11% increase in prevalence of cognitive dysfunction (defined as a score of four or less in a 6-Item Screener, a test of global cognitive function).^100^ Hemodialysis patients are three times more likely to have severe cognitive impairment than age-matched non-dialysis patients even after adjustment for clinical differences, with prevalence rates of 37%.^101^ Although vascular pattern cognitive impairment with executive dysfunction is typical^102^ and several studies link cognitive impairment in CKD to SVD,^103^ other studies suggest that the relationship between CKD and cognitive dysfunction may be independent of SVD.^104^ Neural progenitor cells and the glymphatic system, which are important in Alzheimer disease pathogenesis, have also been proposed to be involved in CKD-associated cognitive impairment.^105^ Multimorbidity itself has been shown to accelerate cognitive decline,^106^ and this effect may be amplified in a post-stroke setting.

## Conclusions and future research directions

Using CKD as an example, we aimed to highlight the impact of multimorbidity on stroke risk, mechanisms, severity, functional recovery, and risk of recurrence or death. As the global burden of CKD rises,^34^ so too will its prevalence in stroke survivors and potential contribution to stroke mechanisms through hypertension and acceleration of atherosclerosis. Patients with proteinuric kidney disease and those who are dialysis-dependent should be recognized as being particularly high-risk for cerebrovascular events and should be given appropriate clinical and research prioritization.

While CKD may share risk factors or certain treatment strategies with stroke, it should still merit special consideration in the design or implementation of stroke prevention and recovery programs. The underlying cause of the kidney disease should also be taken into account, and prevention or treatment plans should be individualized accordingly. Currently, one must cross-reference multiple disparate CKD or stroke guidelines in order to be appropriately guided in the prevention and management of stroke in CKD.^107^ Given the increasing prevalence of overlapping conditions such as CKD, there is a need to standardize or integrate clinical practice guidelines to guide real-world decision-making in frequently multimorbid patients.^108^

Dedicated CKD-specific or -enriched trials are required to expand the evidence base for integrated guidelines. Few cerebrovascular trials to date have reported baseline renal function and many have excluded CKD patients entirely.^30^ Pragmatic trial design may offer advantages over traditional trial design for CKD or other multimorbid patient groups. We have outlined our recommendations for key research priorities in CKD and multimorbidity in Table 1, several of which aim to establish the safety and efficacy of many standard treatments in the CKD population that have not previously been demonstrated.

**Table 1.** Selected research priorities for CKD and multimorbidity in stroke

Research agenda for CKD
Determine the role of genetic factors in the etiology of stroke in CKD.
Determine if there are novel risk factors that contribute to stroke risk in patients with proteinuria.
Regular audit and monitoring of times to acute treatment and rates of treatment in patients with CKD versus those with normal renal function.
Evaluate the efficacy and safety of IV thrombolysis and thrombectomy in CKD.
Investigate potential novel mechanisms by which CKD may increase the severity of stroke event and the risk of early recurrence, such as the nitric oxide deficiency hypothesis.
Determine the role for antiplatelet treatment in primary stroke prevention in CKD.
Determine when anticoagulation should be used in dialysis patients and which agent has the greatest safety and efficacy.
Clarify whether carotid stenting or endarterectomy are superior in CKD.
Investigate the mechanisms of cognitive decline in CKD and chart its natural history.
Research agenda for multimorbidity
Develop a consensus definition for multimorbidity in stroke and determine the best tool to measure it in this setting.
Design and implement care pathways and programs that accommodate patient preferences and priorities.
Explore the short-term impact of multimorbidity on acute stroke care.
Explore the longer-term impact of multimorbidity on stroke rehabilitation services, recurrence risk, and mortality as well as potential predictors of worse outcomes.
Creation of integrated, collaborative stroke guidelines to reduce fragmented care.
Investigate the impact of treatment burden and polypharmacy on multimorbid stroke survivors.

However, research is needed to explore multimorbidity in stroke more broadly. Detailed examination of individual comorbidities, as well as comorbidity clusters and overall multimorbidity, on long-term stroke recurrence by stroke subtype is warranted. Few studies to date, example, have examined the long-term impact of multimorbidity in stroke with its implications for stroke rehabilitation, secondary prevention, and outcomes as well as non-stroke-related care and outcomes. Interdisciplinary collaboration will be central to the redesign of stroke healthcare to adapt to the increasing complexity of multimorbid patients.
